# Is Oxygen Uptake Measurement Enough to Estimate Energy Expenditure During High-Intensity Intermittent Exercise? Quantification of Anaerobic Contribution by Different Methods

**DOI:** 10.3389/fphys.2018.00868

**Published:** 2018-07-09

**Authors:** Valéria L. G. Panissa, David H. Fukuda, Renan S. Caldeira, Jose Gerosa-Neto, Fabio S. Lira, Alessandro M. Zagatto, Emerson Franchini

**Affiliations:** ^1^School of Physical Education and Sport, University of São Paulo, São Paulo, Brazil; ^2^School of Kinesiology and Physical Therapy, University of Central Florida, Orlando, FL, United States; ^3^Exercise and Immunometabolism Research Group, Department of Physical Education, São Paulo State University, São Paulo, Brazil; ^4^São Paulo State University, São Paulo, Brazil; ^5^Australian Institute of Sport, Canberra, ACT, Australia

**Keywords:** oxygen deficit, oxygen uptake post exercise, blood lactate, aerobic metabolism, high intensity exercise

## Abstract

**Purpose:** The aim of the present study was to compare the contributions of the anaerobic pathway as determined by two different methods and energy expenditure during a typical high-intensity intermittent exercise (HIIE) protocol.

**Methods:** A descriptive research design was utilized in which thirteen physically active men performed six experimental sessions consisting of an incremental test (session 1), submaximal tests at 40, 50, 60, 70, 75, 80, 85, 90% of velocity associated with maximum oxygen uptake (v$\dot{V}$O_2max_) with two intensities per session (sessions 2–5), and the HIIE protocol (session 6; 10 efforts of 1 min at v$\dot{V}$O_2max_ interspersed by 1 min of passive recovery). The estimation of anaerobic energy system contribution was calculated by: (a) the excess post-exercise oxygen consumption plus delta lactate method and (b) the accumulated oxygen deficit method using the difference between predicted oxygen demand from the submaximal tests of varying intensities and accumulated oxygen uptake during HIIE. Estimation of aerobic energy system contribution was calculated through the measurement of oxygen consumption during activity. Total EE during the entire HIIE protocol (efforts + recovery) and for the efforts only were calculated from each method.

**Results:** For efforts + recovery and efforts only, anaerobic contribution was similar for both methods, and consequently total EE was also equivalent (*p* = 0.230 for both comparisons). During efforts + recovery, aerobic:anaerobic energy system contribution was (68 ± 4%: 32 ± 4%), while efforts only was (54 ± 5%: 46 ± 5%) with both situations demonstrating greater aerobic than anaerobic contribution (*p* < 0.001 for both).

**Conclusion:** Anaerobic contribution seems to be relevant during HIIE and must to be taken into account during total EE estimation; however, the type of method employed did not change the anaerobic contribution or total EE estimates.

## Introduction

High-intensity intermittent training is considered an efficient strategy to control or decrease fat mass (Trapp et al., 2008; Panissa et al., 2016) that may be superior to moderate-intensity continuous training. The superior benefits of high-intensity intermittent training over moderate-intensity continuous training have included protocols matched for energy expenditure (EE) (Trapp et al., 2008) or mechanical work (Higgins et al., 2016), and even when high-intensity intermittent training is performed with lower volume (Tremblay et al., 1994). However, a recent meta-analysis (Keating et al., 2017) showed no difference in fat loss reduction between intensities.

As EE is an important variable to consider from a weight management perspective (Keating et al., 2017), longitudinal studies aiming to investigate fat mass reduction during high-intensity intermittent training have used oxygen uptake to estimate EE (Tjønna et al., 2008; Trapp et al., 2008; Hwang et al., 2016; Kong et al., 2016). However, estimation via oxygen uptake only likely neglects the contribution of the anaerobic energy system, which can, in turn, underestimate EE during high-intensity intermittent exercise (HIIE). Consequently, if only the oxygen uptake measurement is considered to match EE between high- and moderate-intensity protocols, the results could be biased.

Greater appetite suppression and excess post-exercise oxygen consumption (EPOC) concomitant with higher EE following HIIE are two potential hypotheses for explaining the superior benefits of fat mass reduction following high-intensity intermittent training (Trapp et al., 2008; Boutcher, 2011). The investigation of both hypotheses (aiming to analyze post-exercise oxygen consumption or appetite) typically match or at least report EE because the main outcomes are intensity-dependent, or potentially related to differences in EE or other variables, such as duration or total work done (Deighton et al., 2013; Bailey et al., 2015; Beaulieu et al., 2015; Howe et al., 2016; Panissa et al., 2016; Tucker et al., 2016; Islam et al., 2017). Most studies aiming to analyze EPOC (Tucker et al., 2016; Islam et al., 2017), or appetite (Deighton et al., 2013; Bailey et al., 2015; Beaulieu et al., 2015; Howe et al., 2016), have used the acute evaluation of EE during HIIE based on oxygen uptake.

Estimating the anaerobic contribution to EE is more difficult, and until recently there was no gold standard method (Li et al., 2015). Furthermore, the estimation methods that exist have limitations especially because anaerobic metabolism is often examined using indirect variables (Gastin, 2001; Li et al., 2015). There are two non-invasive main methods of estimating the anaerobic contribution, one of which utilizes changes in lactate to represent the glycolytic contribution and the fast phase of EPOC to represent the phosphagen contribution (EPOC + [La^-^]) (Margaria et al., 1933; di Prampero and Ferretti, 1999). The other method utilizes the concept of oxygen deficit to determine anaerobic contribution (Medbo et al., 1988). This approach examines the difference in the required (theoretical) oxygen uptake demand and the oxygen consumed, which is considered the oxygen deficit derived from anaerobic pathways (i.e., ATP-PCr and glycolysis) (Medbo et al., 1988). The results of both anaerobic energy system estimation methods can be reported in oxygen equivalents that can be combined with the commonly calculated contributions of the oxidative energy system.

The knowledge of the individual energy system contributions during high-intensity intermittent training, which has been shown to improve physical fitness (Milanovic et al., 2015), glycemic control (Wormgoor et al., 2017), lipid profiles, and blood pressure (Cooper et al., 2016), might enhance the understanding of the long-term effects of metabolic adaptations and aid in training program design. Thus, the aim of the present study was to investigate EE during a very common HIIE protocol (1 min effort at v$\dot{V}$O_2max_:1 min passive recovery) including the anaerobic contribution estimated by the EPOC + [La^-^] and oxygen deficit methods. Our hypothesis was that EE may be underestimated when only considering oxygen uptake and that the methods used in the present study would match in terms of energy system contribution and total EE.

## Materials and Methods

### Experimental Design

**FIGURE 1:**
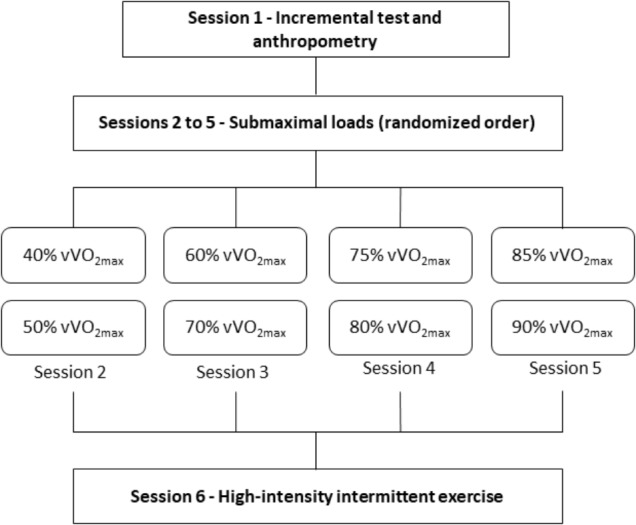
Graphical representation of the study design. v$\dot{V}$O_2max_ = velocity associated with maximum oxygen uptake.

The subjects completed six experimental sessions separated by at least 48 h. During the session 1, anthropometric measurements and a maximal oxygen uptake ($\dot{V}$O_2max_) test on a treadmill were conducted. During sessions 2–5, the participants were submitted to submaximal intensities with 7 min of duration at each velocity (40, 50, 60, 70, 75, 80, 85, 90% of velocity corresponding to $\dot{V}$O_2max_ [v$\dot{V}$O_2max_]) exercising at two intensities per session separated by 30 min of recovery, which used to estimate EE, and during session 6, they completed the HIIE protocol (**Figure 1**).

### Subjects

Thirteen physically active men study (25 ± 5 years; 173.3 ± 8.4 cm and 73.5 ± 10.7 kg) participated in the study. We calculated a sample size required based on a previous study (Miyagi et al., 2017) in which to achieve a power of 0.85 comparing both kinds of methods to estimate the anaerobic contribution a sample size required was 12 participants. The subjects were recruited from a University campus using flyers advertising the investigation. They were included in the study if they reported no health problems and/or neuromuscular disorders that could affect the ability to complete the study protocol. Moreover, the participants were free of drugs or ingestion of nutritional supplements during the period of the study. Participants took part in the study voluntarily after being informed of the procedures, risks, and benefits and signed a consent form. This study was approved by the local ethics committee (CAAE 53297815.8.0000.5204).

### Incremental Test

The participants performed an incremental test until volitional exhaustion on the treadmill (Inbramed-ATL, Brazil). Following a 5-min warm-up completed at 5 km/h, the initial velocity was then set at 8 km/h and increased by 1 km/h every 2 min (Lira et al., 2017). Verbal encouragement was given during the test, and subjects were instructed to perform the test until they could no longer continue. Breath-by-breath oxygen uptake ($\dot{V}$O_2_) was measured (Model Quark PFT Ergo – Cosmed – Rome) throughout the test and $\dot{V}$O_2max_ was considered the highest $\dot{V}$O_2_ attained in the final 30 s of a given stage when it coincided with a change of less than 2.1 mL/kg/min despite increases in treadmill velocity. The velocity associated with $\dot{V}$O_2max_ (v$\dot{V}$O_2max_) was defined as the running velocity at which $\dot{V}$O_2max_ was attained.

### High-Intensity Intermittent Exercise

Participants performed a warm-up at 50% of v$\dot{V}$O_2max_ for 5-min, and after a 2-min rest interval they started the HIIE protocol, which consisted of 10 1-min efforts completed at v$\dot{V}$O_2max_ separated by 1-min of passive recovery on the treadmill. Breath-by-breath $\dot{V}$O_2_ was measured throughout the HIIE protocol and for 6 min post-exercise. Moreover, blood from the ear lobe was collected via capillary tube at rest and immediately after each effort to analyze lactate concentration using an electrochemical analyzer (Yellow Spring 1500 Sport, Yellow Springs, United States). The standard error of lactate measurement with the electrochemical analyzer is 2%.

### Energy Expenditure

The overall EE was estimated during HIIE and corresponded to the sum of the contributions of the oxidative and anaerobic energy systems as used in other investigations (Franchini et al., 2013; Julio et al., 2017). In order to have a better understanding of energy system contribution during HIIE, both the overall energy demand of exercise (efforts + recovery) and the energy demand during only the efforts were considered (Milioni et al., 2017). Moreover, we analyzed the overall HIIE protocol (effort or effort + recovery) as well as each of the 10 individual bouts (each effort or each effort + recovery).

The contribution of the oxidative energy system was estimated by subtracting the $\dot{V}$O_2_-time integral during rest (i.e., resting $\dot{V}$O_2_ × time) from the $\dot{V}$O_2_-time integral during overall exercise (effort + recovery) and only during effort in the analysis of effort isolated, using the trapezoidal method. For the anaerobic energy system estimation, two methods based on the evaluation of EPOC + [La^-^] (Margaria et al., 1933; di Prampero and Ferretti, 1999) and oxygen deficit (O_2deficit_) (Medbo et al., 1988) were used.

To determine the anaerobic energy system contribution for the O_2deficit_ method, linear regression from the oxygen demand and exercise intensity values during the eight submaximal v$\dot{V}$O_2max_ trials (40, 50, 60, 70, 75, 80, 85, 90%) was used to estimate the oxygen demand corresponding to 100% of v$\dot{V}$O_2max_ (Medbo et al., 1988; Bertuzzi et al., 2010). To determine the oxygen deficit, the estimated oxygen uptake at 100% v$\dot{V}$O_2max_ was considered the required oxygen uptake for each HIIE effort and the measured oxygen uptake during each effort was subtracted from this value and considered the anaerobic contribution. This value was summed up with the oxygen uptake during each effort (effort only estimate) and with the oxygen uptake during each effort plus the oxygen uptake measured during the recovery (efforts + recovery estimate).

Comparatively, the anaerobic energy system contribution using the EPOC + [La^-^] was determined from the individual contributions of the phosphagen and glycolytic energy systems. The phosphagen energy system contribution was assumed as the sum of $\dot{V}$O_2_-time integral during the HIIE recovery periods (ΣEPOC). The $\dot{V}$O_2_-time integral during the HIIE recovery periods was used due to the inability to identify the fast component of EPOC as originally proposed (Margaria et al., 1933) and considering that 1-min recovery between efforts is likely predominantly devoted to the reestablishment of creatine phosphate stores (Bogdanis et al., 1995). However, fast component of EPOC (i.e., estimated using $\dot{V}$O_2_ kinetics as the product of $\dot{V}$O_2_ amplitude and tau using a mono-exponential fit) was calculated for the last effort (Milioni et al., 2017) and it was observed a time constant of 66.5 ± 10.1 s and amplitude of 2678.4 ± 390.2 mL/min. The glycolytic energy system contribution was calculated using the sum of the differences between lactate concentrations before effort 1 and immediately after each of the ten efforts ([ΔLa^-^]). Subsequently, [ΔLa^-^] was converted to oxygen equivalents assuming the accumulation of 1 mmol/L [La^-^] is equivalent to 3 mL O_2_/kg of body mass (di Prampero and Ferretti, 1999).

Because the O_2deficit_ method provides anaerobic and aerobic estimation, without division of the anaerobic energy systems (i.e., phosphagen and glycolytic), the EE from the phosphagen and glycolytic systems from the EPOC + [La^-^] were summed together to allow for direct comparisons. For the efforts + recovery and efforts only calculations, the anaerobic energy system contributions were equivalent because the aerobic energy system was not considered during recovery. All oxygen equivalents were converted to energy equivalents assuming 20.92 kJ for each 1 L of O_2_ utilized (Gastin, 2001) prior to EE evaluation.

### Statistical Analysis

The data were analyzed using Statistica (version 10) and results are presented as mean and standard deviation. The normality of data was checked by the Shapiro–Wilk test. The $\dot{V}$O_2_ estimated for 100% v$\dot{V}$O_2max_ and $\dot{V}$O_2max_ attained in incremental test, mean of O_2deficit_ and average EPOC after each effort, total EE estimated from the EPOC + [La^-^] and O_2deficit_ methods were compared via paired student *t*-test. In the overall HIIE protocol analysis, the comparison of energy system contribution by different methods was done by two-way [method (EPOC + [La^-^] vs. O_2deficit_) × energy system contribution (aerobic vs. anaerobic)] ANOVA with repeated measurements in the second factor for the efforts + recovery and efforts only calculations. Moreover, an individual bout comparison of the relative energy system contributions during each effort of the HIIE protocol was conducted using a three-way [method (EPOC + [La^-^] vs. O_2deficit_) × energy system contribution (aerobic vs. anaerobic) × effort number (1–10)] with repeated measurements. When a significant difference was observed, a Tukey *post hoc* test was applied. Statistical significance was set at *p* < 0.05. Partial eta squared ($\eta_{p}^{2}$) was calculated to determine the effect size for ANOVAs, using 0.0099, 0.0588, and 0.1379 as small, medium, and large effect sizes (Cohen, 1988). Effect sizes for the student *t*-tests were calculated using Cohen’*d*: <0.2 – trivial; >0.2 and <0.6 – small; >0.6 and <1.2 – moderate; >1.2 and <2.0 – large; >2.00 and <4.0 – very large; <4.0 – nearly perfect (Hopkins, 2015). Ninety-five percent confidence intervals (95% CI) were calculated for all descriptive variables.

## Results

The **Table 1** presents the performance of participants during the incremental test, the distance ran during the HIIE protocol, and relevant information from the anaerobic energy system contribution estimation methods.

**Table 1:** Performance and parameter values for estimating the energy expenditure during the incremental test and HIIE protocol.

**Incremental test**
$\dot{V}$O_2max_ (mL/kg/min)	52.8 ± 3.5 (50.7 to 54.9)
$\dot{V}$O_2max_ (L/min)	4.0 ± 0.5 (3.7 to 4.3)
v$\dot{V}$O_2max_ (km/h)	13.7 ± 1.2 (13.5 to 13.8)
**High-intensity intermittent exercise**	
Distance ran (km)	2.3 ± 0.2 (2.2 to 3.0)
**EPOC + [La^-^] parameters**	
EPOC average after each effort (L/min)	2.3 ± 0.3 (2.1 to 2.5)
ATP-PCr contribution all efforts (kJ)	422.4 ± 71.7 (379.0 to 465.7)
Δ[La^-^] overall exercise (mmol/L)	5.6 ± 1.3 (4.8 to 6.4)
[La^-^]O_2_ equivalent (L)	1.3 ± 0.3 (1.1 to 1.5)
Glycolytic contribution (kJ)	26.9 ± 6.7 (22.8 to 30.9)
**O_2deficit_ parameters**	
$\dot{V}$O_2_ estimated (L/min)	4.6 ± 0.6 (4.2 to 4.9)
$\dot{V}$$\dot{V}$O_2_ consumed (L/min)	2.6 ± 0.4 (2.4 to 2.9)
O_2deficit_ average after each effort (L/min)	1.9 ± 0.3 (1.7 to 2.2)
R^2^ from 100% v$\dot{V}$O_2max_ estimation	0.92 ± 0.05 (0.9 to 1.0)


*Presented as mean, standard deviation and 95% confidence interval.*

There was no difference between mean O_2deficit_ and EPOC after each effort (*t*_12_ = 0.49; *p* = 0.633; *d* = 0.22 [small]). The $\dot{V}$O_2_ estimated for 100% v$\dot{V}$O_2max_ was greater than $\dot{V}$O_2max_ attained in incremental test (*t*_12_ = 4.81; *p* < 0.001; *d* = 1.08 [large]).

### Overall HIIE Protocol Analysis (Effort or Effort + Recovery)

No differences were found between the O_2deficit_ and EPOC + [La^-^] methods of estimating overall EE for the efforts + recovery (*t*_12_ = 1.26; *p* = 0.230; *d* = 0.234 [small]) and efforts only (*t*_12_ = 1.26; *p* = 0.230; *d* = 0.347 [small]) comparisons (**Table 2**).

**Table 2:** Absolute and percentage contribution of each energy system during HIIE estimated by two methods and reported for efforts + recovery and efforts only.

		Energy contribution (%)	Energy contribution (kJ)	Total energy expenditure (kJ)
**Efforts + recovery**
O_2deficit_	Aerobic	69.2 ± 5.6^∗^ (65.6 to 72.9)	918.2 ± 148.2^∗^ (828.7 to 1007.7)	1323.8 ± 148.0 (1234.3 to 1413.2)
	Anaerobic	30.8 ± 5.6 (27.1 to 34.4)	405.5 ± 81.2 (356.4 to 454.6)	
EPOC + [La^-^]	Aerobic	67.1 ± 0.6^∗^ (66.8 to 67.5)	918.2 ± 148.2^∗^ (828.7 to 1007.7)	1367.6 ± 220.7 (1234.2 to 1500.9)
	Anaerobic	32.9 ± 0.6 (32.5 to 33.2)	449.3 ± 73.2 (405.0 to 493.6)	
**Efforts only**
O_2deficit_	Aerobic	55.1 ± 6.7^∗^ (51.1 to 59.1)	495.8 ± 75.6^∗^ (448.2 to 543.4)	901.3 ± 99.3 (841.3 to 961.4)
	Anaerobic	44.9 ± 6.7 (40.9 to 48.9)	405.5 ± 81.2 (356.4 to 454.6)	
EPOC + [La^-^]	Aerobic	52.5 ± 1.3^∗^ (51.7 to 53.3)	495.8 ± 75.6^∗^ (448.2 to 543.4)	945.1 ± 149.9 (854.5 to 1035.7)
	Anaerobic	47.5 ± 1.3 (46.7 to 48.3)	449.3 ± 73.2 (405.1 to 493.6)	


*Values are mean ± SD and 95% of confidence interval; ^∗^higher than anaerobic contribution (*p* < 0.05).*

From the two way analysis of variance (method vs. energy system contribution), main effects for energy system contribution were found for the efforts + recovery when examining the absolute values (*F*_1,12_ = 187.7; *p* < 0.001; $\eta_{p}^{2}$= 0.918 [large]) and the percentage values (*F*_1,12_ = 219.81; *p* < 0.001; $\eta_{p}^{2}$= 0.948 [large]) with aerobic contribution being greater than anaerobic contribution in both cases (absolute: *p* < 0.001; percentage: *p* < 0.001). Similar results were found for the efforts only comparison, with main effects for energy system contribution when examining the absolute values (*F*_1,12_ = 14.16; *p* = 0.003; $\eta_{p}^{2}$= 0.541 [large]) and the percentage values (*F*_1,12_ = 15.05; *p* = 0.002; $\eta_{p}^{2}$= 0.557 [large]) with aerobic contribution being greater than anaerobic contribution (absolute: *p* = 0.003; percentage: *p* = 0.002).

For the effort and recovery analysis, there was no main effect for method when absolute (*F*_1,12_ = 1.60; *p =* 0.230; $\eta_{p}^{2}$= 0.118 [moderate]) and relative values (*F*_1,12_ = 0.00; *p =* 1.00; $\eta_{p}^{2}$= 0.112 [moderate]) were considered, neither interaction between method and energy system contribution for absolute (*F*_1,12_ = 1.60; *p =* 0.230; $\eta_{p}^{2}$= 0.118 [moderate]) and relative values (*F*_1,12_ = 1.51; *p* = 0.242; $\eta_{p}^{2}$= 0.112 [moderate]).

For the effort only analysis, there was no main effect for method when absolute (*F*_1,12_ = 1.60; *p* = 0.230; $\eta_{p}^{2}$= 0.118 [moderate]) and relative values (*F*_1,12_ = 0.00; *p =* 1.00; $\eta_{p}^{2}$= 0.136 [moderate]) were considered. Furthermore, no interaction between method and energy system contribution for absolute (*F*_1,12_ = 1.60; *p =* 0.230; $\eta_{p}^{2}$= 0.118 [moderate]) and relative values (*F*_1,12_ = 1.51; *p =* 0.242; $\eta_{p}^{2}$= 0.136 [moderate]) was found.

### Individual Bout Analysis (Each Effort or Each Effort + Recovery)

**FIGURE 2:**
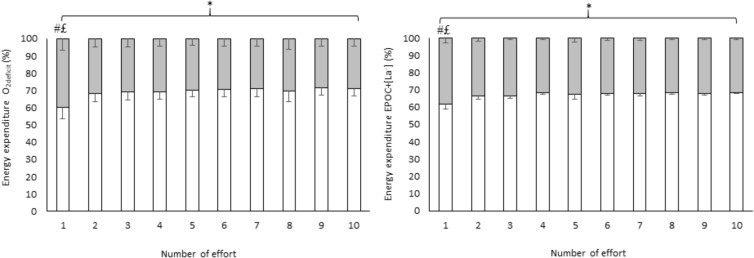
Effort by effort comparison of energy system contribution during HIIE estimated by two methods calculated using efforts + recovery. Values are mean ± SD; white = aerobic contribution; gray = anaerobic contribution; ^∗^higher aerobic contribution than anaerobic contribution (*p* < 0.001); ^#^lower aerobic contribution compared with aerobic contribution in all subsequent efforts (*p* < 0.001); £higher anaerobic contribution compared with anaerobic contribution in all subsequent efforts (*p* < 0.001). Ninety-five percent confidence intervals of each effort + recovery are presented in Supplementary Table S1.

The effort by effort comparison when considering EE from the efforts + recovery (**Figure 2**) demonstrated an energy system contribution main effect (*F*_1,12_ = 861.9; *p* < 0.001; $\eta_{p}^{2}$= 0.986) with aerobic contribution being greater than anaerobic contribution (*p* < 0.001) There was also energy system contribution by effort number interaction (*F*_9,108_ = 346.20; *p* < 0.001; $\eta_{p}^{2}$= 0.693 [large]), with effort 1 showing lower aerobic contribution and greater anaerobic contribution than all subsequent efforts (*p* < 0.001 for all comparison) and greater aerobic contribution than anaerobic contribution within each effort (*p* < 0.001 for all comparisons). There was also an interaction for method, energy system and number of effort (*F*_9,108_ = 6.37; *p* < 0.001; $\eta_{p}^{2}$= 0.346 [large]), but Tukey *post hoc* tests indicated similar differences to those found with the energy system by number of effort interaction. There was no main effect for method (*F*_1,12_ = 0.00; *p* = 1.00; $\eta_{p}^{2}$ = 0.001 [small]), number of effort (*F*_9,108_ = 0.00; *p* = 1.00; $\eta_{p}^{2}$ = 0.001 [small]) method and number of effort interaction (*F*_9,108_ = 5.58; *p* = 1.00; $\eta_{p}^{2}$ = 0.001 [small]) and method and energy system contribution interaction (*F*_9,108_ = 2.52; *p* = 0.138; $\eta_{p}^{2}$ = 0.173 [large]).

**FIGURE 3:**
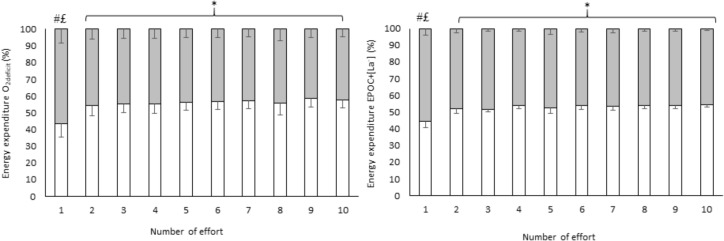
Effort by effort comparison of energy system contribution during HIIE estimated by two methods calculated using efforts only. Values are mean ± SD; white = aerobic contribution; gray = anaerobic contribution; ^∗^higher aerobic contribution than anaerobic contribution (*p* < 0.001); ^#^lower aerobic contribution compared with anaerobic contribution in the first effort; ^£^higher anaerobic contribution compared with anaerobic contribution in all subsequent efforts (*p* < 0.001). Ninety-five percent confidence intervals of each effort are presented in Supplementary Table S2.

According to the three-way analysis (method × energy system contribution × effort number), the effort by effort (**Figure 3**) comparison considering EE from the efforts only demonstrated main effect of energy system contribution (*F*_1,12_ = 30.7; *p* = 0.001; $\eta_{p}^{2}$= 0.998 [large]) with aerobic contribution being greater than anaerobic contribution and (*p* = 0.001), an energy system contribution by effort number interaction (*F*_9,108_ = 56.72; *p* < 0.001; $\eta_{p}^{2}$= 0.703 [large]); however, there was lower aerobic contribution than anaerobic contribution in the effort 1 (*p* < 0.001) and greater aerobic contribution than anaerobic contribution in efforts 2 through 10 (*p* < 0.001 for all comparisons). Additionally, the anaerobic contribution in the first effort was greater than all subsequent efforts (*p* < 0.001 for all comparisons). There was also an interaction for method, energy system and number of effort (*F*_9,108_ = 3.97; *p* < 0.001; $\eta_{p}^{2}$= 0.248 [large]), but Tukey *post hoc* tests indicated similar differences to those found with the energy system by number of effort interaction. There was no main effect for method (*F*_1,12_ = 0.04; *p* = 0.840; $\eta_{p}^{2}$ = 0.003 [small]), number of effort (*F*_9,108_ = 0.00; *p* = 0.872; $\eta_{p}^{2}$= 0.039 [small]) and method and energy system contribution interaction (*F*_9,108_ = 2.91; *p* = 0.113; $\eta_{p}^{2}$ = 0.195 [moderate]), or method and number of effort interaction (*F*_9,108_ = 0.49; *p* = 0.872; $\eta_{p}^{2}$ = 0.039 [small]).

## Discussion

The main findings of present study were that the use of only $\dot{V}$O_2_ underestimates total EE during HIIE and that the type of method used did not change this result. This is the first study to evaluate the anaerobic and aerobic demands on a typical HIIE protocol (10 × 1 min at v$\dot{V}$O_2max_:1 min passive recovery), which has been widely utilized due to positive results on health markers and feasibility for healthy (Little et al., 2010; Lira et al., 2017), and overweight/obese populations (Gillen et al., 2013; Smith-Ryan et al., 2015; Sawyer et al., 2016).

Generally, the underestimation of EE based only on $\dot{V}$O_2_ measurement occurs because the anaerobic contribution is neglected. Given that the anaerobic participation is small during steady-state exercise this issue does not appear to significantly affect total EE estimation. However, during HIIE, the anaerobic contribution in this study was approximately 30% (efforts + recovery for both methods), demonstrating that if the anaerobic contribution is not considered, there is a considerable underestimation of EE. When we considered just the efforts, the anaerobic contribution reached ∼50%, supporting the clear dependence of anaerobic metabolism to perform the investigated HIIE protocol. This proportion was maintained during all subsequent efforts with only first effort requiring greater anaerobic contribution.

Previous investigations examining EE during high-intensity exercise varied considerably with respect to the protocol examined, including one effort until exhaustion with a determined fixed load (Bertuzzi et al., 2010; Zagatto et al., 2011, 2016) or repeated all out efforts (Franchini et al., 2016; Milioni et al., 2017), while the primary aim was usually the identification of maximal anaerobic capacity (Bertuzzi et al., 2010; Zagatto et al., 2011, 2016; Milioni et al., 2017). For instance, utilizing similar methods to those employed in the current study, Bertuzzi et al. (2010) reported no differences in a single cycling bout until exhaustion at 110% of maximal aerobic power, estimating an anaerobic contribution of ∼63% (time limit 171 ± 39 s) with both methods (O_2deficit_ or EPOC + [La^-^]). Thus, our investigation highlights a unique application of these methods by providing evidence of their equivalence when HIIE is considered.

It is important to consider the main limitations of each method used in the present study. The main limitation of the O_2deficit_ method is the extrapolation of the exercise intensity and oxygen uptake during submaximal loads to supramaximal intensities (Zoladz et al., 1995; Green and Dawson, 1996). However, this limitation was reduced in our study, as the exercise intensity used was equivalent to v$\dot{V}$O_2max_. Thus, further investigations should test if the similarity between these methods are still equivalent when supramaximal exercise bouts are used. Additionally, this method has been the most widely adopted (Li et al., 2015) due to its association with performance in anaerobic tests (Scott et al., 1991), and sensitivity to training (Weber and Scheneider, 2002).

The main limitation of the EPOC_fast_ + [La^-^] method is the assumption that accumulated [La^-^] measured post-exercise appropriately represents the glycolytic energy system contribution, given plasma [La^-^] represents a balance between lactate production and clearance, which may occur in other tissues (Bangsbo, 1998). Moreover, during repeated exercise efforts, this method likely underestimates anaerobic contribution due to the complex interaction of the energy systems during intermittent recovery periods (Trump et al., 1996; Spencer and Gastin, 2001).

In the present study, the contribution of the glycolytic system estimated by [ΔLa^-^] following each effort was just ∼26 kJ (5.6 mmol/L total accumulated). In contrast, the contribution of ATP-PCr system (considering oxygen uptake during recovery between efforts) may be overestimated due to limitations with precisely estimating the fast phase of oxygen uptake after each effort. However, Bogdanis et al. (1995) and Beneke et al. (2002) demonstrated that after 30 s of “all-out” cycling, the half-times of phosphocreatine recovery were 56 ± 7 and 47 ± 12 s, respectively. Considering the association between the fast phase of EPOC and ATP-PCr resynthesis (Margaria et al., 1933), $\dot{V}$O_2_ kinetics data, which are used as proxy indicators of ATP-PC resynthesis, following the final effort in the current study resulted in 2.9 ± 0.6 L of oxygen consumed (amplitude of 2678 ± 390 ml of oxygen consumed and time constant of 66 ± 10 s) which was higher than oxygen uptake after each effort (the fast phase of EPOC).

In addition to the comparison of method used to determine EE, the current findings must be considered within the context of the planning and prescription of HIIE. Strength and conditioning coaches and fitness professionals may use this knowledge of the energetic demands required during a typical HIIE session to support or reinforce the decision-making process involved with training program design. Exercise modality and training status must also be considered. This is especially important because more aerobically trained individuals present faster PCr recovery (Yoshida, 2002) which likely influences oxygen uptake kinetics (Panissa et al., 2014). Considering many variables can be manipulated during HIIE (e.g., exercise and interval intensity, volume, effort-pause ratio, exercise mode) (Buchheit and Laursen, 2013), knowledge of energy system contribution during specific protocols can be used to improve exercise prescription. For example, if we consider that the fast phase of oxygen recovery represents the restoration of phosphocreatine following high-intensity exercise, the utilization of active recovery could completely alter the dynamics of energy system contribution.

## Conclusion

Our data demonstrated that the inclusion of anaerobic contribution in HIIE added ∼30% to total EE and there was no difference between methods employed (the O_2deficit_ and EPOC_fast_ + [la]). Furthermore, there are limitations to estimating anaerobic contribution and the current attempt is limited to the examined HIIE protocol (10 efforts of 1 min at v$\dot{V}$O_2max_ interspersed by 1 min of passive recovery), more accurate analyses will assist in understanding the effects of exercise intensity on variables that depend on EE, such as fat mass, appetite or EPOC. Thus, more precise estimation of the non-oxidative contribution can be developed to understand various interventions, including ergogenic aids, and to improve the understanding of energy system utilization during HIIE protocols. Finally, the current results may need to be confirmed using more invasive or complex methods, such as muscle biopsies or functional magnetic resonance imaging, in an effort to gain a more direct understanding of the changes in muscle metabolites related to the glycolytic and ATP-PCr systems.

## Author Contributions

The study was designed by VP, DF, and EF. Data were collected and analyzed by VP, RC, and JG-N. Data interpretation and manuscript preparation were undertaken by VP, DF, RC, JG-N, FL, AZ, and EF. All authors approved the final version of the paper.

## Conflict of Interest Statement

The authors declare that the research was conducted in the absence of any commercial or financial relationships that could be construed as a potential conflict of interest.
