# Supplementary material for: Is Oxygen Uptake Measurement Enough to Estimate Energy Expenditure During High-Intensity Intermittent Exercise? Quantification of Anaerobic Contribution by Different Methods
Source: Front Physiol. 2018 Jul 9;9:868. doi: 10.3389/fphys.2018.00868 (PMC6046462; doi:10.3389/fphys.2018.00868)
Supplement: Supplementary file 1 [file Data_Sheet_1.DOCX]

**Table 1:** Data of energy system contribution estimated by oxygen deficit (MAOD) or fast phase excess post-exercise oxygen consumption plus lactate concentration (EPOC+ [La^-^]) from individual bout analysis (each effort +recovery).

|  | **AEROBIC** | | **ANAEROBIC** | |
| --- | --- | --- | --- | --- |
| **Effort number** | MAOD (mean ± sd; CI 95%) | EPOC+ [La^-^] (mean ± sd; CI 95%) | MAOD (mean ± sd; CI 95%) | EPOC+ [La^-^] (mean ± sd; CI 95%) |
| **1** | 60.4 ± 6.6 (56.4 to 64.4) | 61.9 ± 3.0 (60.2 to 63.7) | 39.6 ± 6.6 (35.6 to 43.6) | 38.1 ± 3.0 (36.3 to 39.8) |
| **2** | 68.3 ± 4.9 (65.3 to 71.3) | 66.3 ± 1.9 (65.1 to 67.4) | 31.7 ± 4.9 (28.7 to 34.7) | 33.7 ± 1.9 (32.5 to 34.9) |
| **3** | 69.4 ± 4.7 (66.5 to 72.2) | 66.3 ± 1.2 (65.6 to 67.0) | 30.6 ± 4.7 (27.8 to 33.5) | 33.7 ± 1.2 (33.0 to 34.4) |
| **4** | 69.4 ± 4.5 (66.7 to 72.1) | 68.3 ± 0.8 (67.8 to 68.8) | 30.6 ± 4.7 (27.9 to 33.3) | 31.7 ± 0.8 (31.2 to 32.2) |
| **5** | 70.1 ± 4.0 (67.7 to 72.6) | 67.2 ± 2.6 (65.6 to 68.7) | 29.9 ± 4.0 (27.4 to 32.3) | 32.8 ± 2.6 (31.3 to 34.4) |
| **6** | 70.6 ± 4.2 (68.0 to 73.1) | 68.1 ± 1.4 (67.3 to 68.9) | 29.4 ± 4.2 (26.9 to 32.0) | 31.9 ± 1.4 (31.1 to 32.7) |
| **7** | 70.9 ± 4.4 (68.2 to 73.6) | 68.1 ± 1.5 (67.2 to 69.0) | 29.1 ± 4.4 (26.4 to 31.8) | 31.9 ± 1.5 (31.0 to 32.8) |
| **8** | 69.8 ± 6.1 (66.1 to 73.5) | 68.5 ± 1.0 (67.9 to 69.1) | 30.2 ± 6.1 (26.5 to 33.9) | 31.5 ± 1.0 (30.9 to 32.1) |
| **9** | 71.7 ± 5.5 (68.4 to 75.0) | 67.8 ± 1.0 (67.2 to 68.4) | 28.3 ± 5.5 (25.6 to 31.6) | 32.2 ± 1.0 (31.6 to 32.8) |
| **10** | 71.2 ± 4.2 (68.7 to 73.8) | 68.5 ± 0.8 (68.0 to 69.0) | 28.8 ± 4.2 (26.2 to 31.3) | 31.5 ± 0.8 (31.0 to 32.0) |

**Table 2.** Data of energy system contribution estimated by oxygen deficit (MAOD) or fast phase excess post-exercise oxygen consumption plus lactate concentration (EPOC+ [La^-^]) from individual bout analysis (each effort only).

|  | **AEROBIC** | | **ANAEROBIC** | |
| --- | --- | --- | --- | --- |
| **Effort number** | MAOD (mean ± sd; CI 95%) | EPOC+ [La-] (mean ± sd; CI 95%) | MAOD (mean ± sd; CI 95%) | EPOC+ [La-] (mean ± sd; CI 95%) |
| **1** | 43.5 ± 8.3 (38.5 to 48.5) | 44.5 ± 4.0 (40.5 to 47.0) | 56.5 ± 8.3 (51.5 to 61.5) | 55.5 ± 4.0 (51.4 to 57.9) |
| **2** | 54.3 ± 6.0 (50.7 to 58.0) | 51.8 ± 2.7 (49.2 to 53.5) | 45.7 ± 6.0 (42.0 to 49.3) | 48.2 ± 2.7 (45.5 to 49.8) |
| **3** | 55.4 ± 5.5 (52.0 to 58.7) | 51.6 ± 1.6 (50.0 to 52.6) | 44.6 ± 5.5 (41.3 to 48.0) | 48.4 ± 1.6 (46.8 to 49.3) |
| **4** | 55.2 ± 5.4 (51.9 to 58.4) | 53.7 ± 1.7 (52.0 to 54.8) | 44.8 ± 5.4 (41.6 to 48.1) | 46.3 ± 1.7 (44.5 to 47.3) |
| **5** | 56.2 ± 4.9 (53.3 to 59.2) | 52.7 ± 3.7 (49.0 to 54.9) | 43.8 ± 4.9 (40.8 to 46.7) | 47.3 ± 3.7 (43.6 to 49.5) |
| **6** | 56.9 ± 4.8 (53.9 to 59.8) | 53.8 ± 2.2 (51.6 to 55.1) | 43.1 ±4.8 (40.2 to 46.1) | 46.2 ± 2.2 (44.0 to 47.6) |
| **7** | 57.2 ± 4.7 (54.3 to 60.0) | 53.6 ± 2.4 (51.3 to 55.1) | 42.8 ± 4.7 (40.0 to 45.7) | 46.4 ± 2.4 (44.0 to 47.8) |
| **8** | 55.9 ± 7.0 (51.7 to 60.1) | 54.1 ± 1.9 (52.2 to 55.2) | 44.1 ± 7.0 (39.9 to 48.3) | 45.9 ± 1.9 (44.1 to 47.1) |
| **9** | 58.4 ± 5.2 (55.3 to 61.6) | 53.7 ± 1.6 (52.1 to 54.6) | 41.6 ± 5.2 (39.4 to 45.1) | 46.3 ± 1.6 (44.8 to 47.3) |
| **10** | 57.7 ± 4.7 (54.9 to 60.6) | 54.5 ± 1.4 (53.1 to 55.3) | 42.3 ± 4.7 (39.4 to 45.1) | 45.5 ± 1.4 (44.1 to 46.4) |
